# Sleep disorders among patients suffering from road traffic injuries in an urban setting of Vietnam: an exploratory study

**DOI:** 10.1038/s41598-023-38693-7

**Published:** 2023-07-17

**Authors:** Hai Minh Vu, Tung Hoang Tran, Anh Kim Dang, Trong Nang Hoang, Cuong Tat Nguyen, Huong Lan Thi Nguyen, Carl A. Latkin, Cyrus S. H. Ho, Roger C. M. Ho

**Affiliations:** 1 Department of Trauma, Thai Binh University of Medicine and Pharmacy, Thai Binh, 410000 Vietnam; 2 Institute of Orthopaedic and Trauma Surgery, Vietnam-Germany Hospital, Hanoi, 100000 Vietnam; 3 Institute for Preventive Medicine and Public Health, Hanoi Medical University, Hanoi, 100000 Vietnam; 4 Department of Ophthalmology, Thai Binh University of Medicine and Pharmacy, Thai Binh, 410000 Vietnam; 5 Institute for Global Health Innovations, Duy Tan University, Da Nang, 550000 Vietnam; 6 Faculty of Medicine, Duy Tan University, Da Nang, 550000 Vietnam; 7 Bloomberg School of Public Health, Johns Hopkins University, Baltimore, MD 21205 USA; 8 Department of Psychological Medicine, Yong Loo Lin School of Medicine, National University of Singapore, Singapore, 119228 Singapore; 9 Institute for Health Innovation and Technology (iHealthtech), National University of Singapore, Singapore, 119077 Singapore

**Keywords:** Psychology, Health care, Medical research

## Abstract

Sleep quality is an important indicator of treatment outcome for patients with traffic accident injuries. In Vietnam, the impacts of injury on sleep status are usually amplified in urban areas due to disproportionate distribution of mental care services between the city and less developed settings. Our study investigated deterioration in sleep quality and identified associated demographic factors among traffic injury patients in an small urban setting of Vietnam. A cross-sectional study was conducted among 408 patients in one provincial hospital and five district hospitals in Thai Binh, Vietnam from October to December 2018. A structured questionnaire was designed based on 3 standardized scales: Health-related Quality of Life, the Pittsburgh Sleep Quality Index and the Kessler Scale. Face-to-face interviews and medical records were conducted by trained health professionals on patients hospitalized in the Trauma—Orthopedic/Burn Department and Surgery and General Department. About 16.9% of respondents had sleep disturbances, and there was a statistically significant difference between age group (p < 0.01), education level (p < 0.01), and monthly household income (p < 0.01) between participants who with and without sleep disturbances. Furthermore, more than half (50.7%) of respondents sleep less than 5 h per day, while 18.7% of the sampled also reported that the habitual sleep efficiency was below 85%. Current results indicated that people being female, suffering from traumatic brain injury, being comatose at hospitalization, and having higher psychological distress scores were more likely to suffer from sleep problems. Our study is one of the first evidence in Vietnam to assess sleep disturbances in road traffic injury patients and their correlated factors. It is important to identify patients who are at risk of sleep disturbances based on socio-demographic and clinical characteristics, as well as psychological distress status. Therefore, a holistic approach should be taken to include sleep quality and psychological state in the treatment process and outcome assessment for road traffic injury patients.

## Introduction

Road traffic injury is a major cause of fatality worldwide, responsible for 1.3 million deaths per year^1^. In Vietnam, the number of death causing by road traffic injury has increased dramatically within 10 years with more than 18,000 traffic accidents and 8125 deaths in 2018 as reported by the General Statistics Office of Vietnam^2^. Traffic-related injury accounts for 11% of all deaths in Vietnam and is predicted to become one of three leading death causes by 2022^3,4^. High rates of traffic accidents in Vietnam are often attributed to heavy density of vehicles and lack of road safety measures and infrastructure^5,6^. It was estimated that 94.1% Vietnamese people own at least one motorcycle and approximately 90% of the population use motorcycle as their primary vehicle^7,8^. The burden of traumatic injury is present in multi aspects of life, including long-term physical health, psychological state, financial status, job quality and daily functioning productivity^9,10^.

Sleep-related characteristics are recognized worldwide as an important indicator of quality of life among patients. Sleep is a vital component of one’s well-being in maintaining brain function and a wide range of systemic physiology such as metabolism, immune, hormonal, and cardiovascular systems^11,12^. One’s sleep is qualified as “healthy” when it meets sufficient duration and without any sleep disturbances^13^. Sleep disorders, especially insomnia, after road traffic injuries, are commonly attributed to post-traumatic stress disorder^14^. The disruption of brain function due to trauma and a myriad of other psychiatric disturbances as well as environmental, financial factors following hospitalization can result in critical worsening of sleep^15^. Indeed, deterioration in sleep quality has been found to be three times more prevalent among traumatic injury patients compared to the general population, approximately 60% of which are long-term or lifelong^16^. Specifically, the previous evidence indicated that a significant prevalence of sleep–wake disturbances can be observed from 6 to 18 months after traumatic injury^17,18^. Moreover, a previous meta-analysis by Mathias has indicated that 50% of injury patients had sleep disturbances, and 25–29% of them were diagnosed with sleep disorders. More recently, in another review by Montgomery^19^, the authors reported that there were 71.7% of post-injury patients have insomnia symptoms and about 27.0% of them have diagnosed with sleep disturbance^19^. Furthermore, some common sleep such as insomnia, obstructive sleep apnea, post-traumatic hypersomnia, narcolepsy and nightmares also indicated^20–23^. Numerous negative health outcomes and disorders have been linked to psychological changes related to sleep disorders. Sleep disturbances directly affect functionality, cognition and mood of patients by upsetting stress hormones^24^. A wealth of evidence has revealed up to 50 psychosocial problems associated with sleep disruption such as emotional distress, deficits in cognitive and memory and performance efficiency, risk of depression and suicidal thoughts^25–28^. In spite of devastating impacts on patients, sleep quality and psychological impacts in general are rarely considered a part of treatment for hospitalized patients across all types of injury.

In Vietnam, there is a disproportionate distribution of mental care services between residential settings. Healthcare services in small urban areas are generally poorer in quantity, quality and delivery than in more developed settings such as big cities and metropolis areas. Disparities in service are due to poorer-trained workforce and under-invested health facilities^29,30^. Moreover, mental health services available are often provided by the private sector and not included in the national health insurance scheme, making mental care highly unaffordable and inaccessible to a non-affording small urban residents^31,32^. Compared to metropolitan residents, common socioeconomic demographic characteristics found among small urban citizens are the lower level of education, labor-heavier work, lower average income and more restricting working schedules^32,33^. Underestimation of mental health problems and a low budget for healthcare services are prime barriers toward mental health care utilization in small urban settings. As a result, consideration for mental issues, specifically sleep disorders is not offered or deliberately excluded in a treatment plan^34,35^.

In the limited amount of evidence provided on impacts of traffic injuries on quality of life, no research has focused on sleep quality and prevalence of sleep disorders as their main objective. These characteristics were either discussed briefly or merely presented in previous studies^36,37^. Acknowledging a lack of practice and research on sleep disorders among patients with traffic accident injuries in Vietnam, our study aimed to assess sleep quality, sleep disorder patterns and demographic factors associated with sleep deterioration among Vietnam traffic injury patients as well as proposed interventions for clinical treatment and public health prevention measures.

## Methods

### Study settings and sampling technique

We performed a cross-sectional study at the Trauma—Orthopedic/Burn and Surgery department of Thai Binh Hospital (one provincial hospital) and the General Department of five district hospitals including Kien Xuong, Hung Ha, Dong Hung, Quynh Phu, Thai Thuy in Thai Binh province, Vietnam from August 2018 to August 2019. The convenience sampling technique was used to recruit participants who met the following criteria: (1) aged at least 18 years old; (2) undergoing treatment from mentioned hospitals; (3) hospitalized for traffic accidents and (4) able to answer questions from data collectors. We excluded patients who had severe injuries. In the above-mentioned hospitals, before patients completed the treatment process and were discharged from the hospital, they were approached and invited to participate in the study. Participants were clearly informed of purposes, advantages and drawbacks of the study and signed written consents before answering the questionnaire. At the end of data collection, a total of 408 participants took part in the interview.

### Measurements and instruments

In this study, the research instrument was developed based on a standard procedure. Firstly, we conducted a review to identify gaps and importance aspects of the study topic. Secondly, an instrument that coverd all facets of topics of interest was developed. Then, several injury experts, psychological experts, policymakers, and health services providers were invited to translate, rephrase, pilot, and shorten the questionnaire. Finally, a structured questionnaire with three main components was developed for face-to-face interview, including (1) Demographic characteristics, (2) Health-related Quality of Life (HRQOL) and Psychological distress, (3) Sleep disorders (The Pittsburgh Sleep Quality Index). Furthermore, for characteristics of injuries, medical records of participants were used to extract the information. Before collecting data process, 50 patients with different socio-economic backgrounds (age, gender, occupation) were invited to pilot the questionnaire. Well-trained experts including healthcare workers from the hospitals were in charge of interviewing patients to ensure the quality of data. We carried out the interviews in a private room in order to secure their confidentiality.

#### Demographic characteristic

Information about age, living area, gender, level of educational, occupation, marital status, and monthly income was collected from the participants. In addition, participants were asked about whether they were inpatients or outpatients.

#### Characteristics of injuries

We imported data from medical records regarding types of traffic injuries and divided them into 8 main categories: soft-tissue, traumatic brain injury, oral and facial, spinal cord injury, chest, hand, fracture, and multiple injuries. Traumas of oral and facial areas were soft tissue injuries, nasal injuries, and fractures. Hand injury included injuries of wrist, hand, and finger (all related structures). Fractures mentioned injuries of limbs (bones of arms, forearms, femurs, kneecaps, shins, and feet).

#### Health-related Quality of Life (HRQOL)

The EuroQol—5 dimensions—5 levels (EQ-5D-5L) scale was used to examine the health-related quality of life (HRQOL) of participants. This tool contained 5 domains of evaluation (Mobility, Self-care, Usual activities, Pain/Discomfort, and Anxiety/Depression) and each domain was rated by a Likert scale (from no problem to severe problem). All domains in the EQ-5D-5L instrument were transformed into an index score using a Vietnamese crosswalk value set^38^. In addition, HRQOL of patients was assessed using EQ-VAS which ranged from 0 (the lowest score of health status) to 100 points (the highest score of health status).

#### Psychological distress

Kessler scale (K6), an instrument used to screen mental health problems, was utilized to measure psychological distress among participants. The scale included six items and each question was scored from 0 to 4, which resulted in a total score from 0 to 24. The cut-off points for having psychological distress was > 5. The Cronbach's alpha was 0.7421^39^.

#### The Pittsburgh Sleep Quality Index (PSQI)

The Pittsburgh Sleep Quality Index (PSQI) was a self-rate questionnaire which was applied to assess the sleep quality and disturbances over a 1-month time interval. The PSQI had 19 questions generating seven components: (1) subjective sleep quality, (2) sleep latency, (3) wake up duration, (4) habitual sleep efficiency, (5) sleep disturbances, (6) use of sleeping procedures, (7) daytime dysfunction over the last month. Each component receives a score from 0 (none during the past month) to 3 (at least three times a week). The scores range from 0 to 21 and dive into 2 groups: Normal sleep quality (0–5 score) and sleep disturbance (higher than 5 score). Participants who had higher scores had worse sleep quality^40^.

### Statistical analysis

Stata version 16 software (Stata Corp. LP, College Station, United States of America) was used to analyze data. Chi-square, Fisher exact tests, and Mann-Whitney tests were used to identify the differences of variables between two self-rated sleep quality groups (normal and sleep disturbance). Multivariate Logistic regression model and Multivariate Tobit regression were utilized to identify factors related to sleep disorders of participants. In this study, the stepwise forward method was utilized to reduce the variables and find the optimal regression models with variables having p-value < 0.2. A p-value < 0.05 is considered statistically significant.

### Ethics approval and consent to participate

The study protocol was reviewed and granted ethics approval by the Institutional Review Board of Thai Binh University of Medicine and Pharmacy (No 7642/HĐĐĐ). All experimental protocols were approved by the Institutional Review Board of Thai Binh University of Medicine and Pharmacy. Informed consent was obtained from all participants. The study was not conducted on participants under 18 years of age. All procedures performed were in accordance with the ethical standards of the institutional and/or national research committee and with the Helsinki Declaration and its later amendments or comparable ethical standards.

## Results

Table 1 shows the socio-economic characteristics of the participants. Among 408 patients participating in the study, 61% (n = 249) of participants were male. The majority of patients had under high school and high school education (81.2%, n = 331). The percentage of participants being employed was 60.8% (n = 248) and 85.3% (n = 348) of participants lived in rural areas. 16.9% (n = 69) of respondents had sleep disturbance.

**Table 1:** Socio-economic characteristics of respondents.

Characteristics	Self-rated sleep quality						
	Sleep disturbance		Normal		Total		p-value
	n	%	n	%	n	%	
Total	69	16.9	339	83.1	408	100	
Age group							
< 30	7	10.1	92	27.1	99	24.3	< 0.01
30–< 45	11	15.9	91	26.8	102	25.0	
45–< 60	19	27.5	86	25.4	105	25.7	
≥ 60	32	46.4	70	20.6	102	25.0	
Gender							
Female	39	56.5	120	35.4	159	39.0	
Male	30	43.5	219	64.6	249	61.0	
Educational level							
Under high school	51	73.9	141	41.6	192	47.1	< 0.01
High school	9	13.0	130	38.3	139	34.1	
Above high school	9	13.0	68	20.1	77	18.9	
Marital status							
Single	19	27.5	94	27.7	113	27.7	< 0.01
Living with spouse/partner	50	72.5	245	72.3	295	72.3	
Employment							
Employed	41	59.4	207	61.1	248	60.8	0.14
Self-employed	10	14.5	74	21.8	84	20.6	
Others	18	26.1	58	17.1	76	18.6	
Living area							
Urban	13	18.8	47	13.9	60	14.7	0.97
Rural	56	81.2	292	86.1	348	85.3	
Types of patients							
In-patents	36	58.1	218	70.1	254	68.1	0.06
Out-patient	26	41.9	93	29.9	119	31.9	

	Mean	SD	Mean	SD	Mean	SD	p-value
Monthly household income (USD)	297.7	250.2	407.2	227.9	388.7	235.1	< 0.01

Note: 1 USD ≈ 22730 VND^41^.

Table 2 describes the health status of traffic accident respondents. Most participants experienced pain or discomfort (98.8%, n = 403), anxiety or depression (90.9%, n = 371), and having problems with usual activities (90.4%, n = 369). Soft-tissue, traumatic brain injury and fracture were the most common injuries among respondents. The body part injuries were mainly limbs (56.1%, n = 229) and head, face, neck (24.5%, n = 100). The average EQ-VAS was 0.66 (SD = 0.26) and EQ-5D-5L index was 0.37 (SD = 0.37). The mean score of psychological distress among patients having sleep disturbance was significantly higher than those having better sleep quality (Mean = 3.8 and 2.3 respectively).

**Table 2:** Health status of traffic accident patients.

Characteristics	Self-rated sleep quality						
	Sleep disturbance		Normal		Total		p-value
	n	%	n	%	n	%	
Health status when hospitalized							
Conscious	67	97.1	321	94.7	388	95.1	0.55
Comatose	2	2.9	18	5.3	20	4.9	
Having problems with							
Mobility	56	81.2	283	83.5	339	83.1	0.64
Self-care	59	85.5	306	90.3	365	89.5	0.24
Usual activities	63	91.3	306	90.3	369	90.4	0.79
Pain/discomfort	68	98.6	335	98.8	403	98.8	1.00
Anxiety/depression	65	94.2	306	90.3	371	90.9	0.30
Types of injuries							
Soft-tissue	21	30.4	92	27.1	113	27.7	0.58
Hand	3	4.4	15	4.4	18	4.4	1.00
Traumatic brain injury	11	15.9	65	19.2	76	18.6	0.53
Oral and facial	2	2.9	22	6.5	24	5.9	0.40
Spinal cord injury	6	8.7	12	3.5	18	4.4	0.10
Chest	3	4.4	9	2.7	12	2.9	0.44
Fracture	21	30.4	122	36.0	143	35.1	0.38
Multiple injuries	3	4.4	15	4.4	18	4.4	1.00
Body part injured							
Head, face, neck	13	18.8	87	25.7	100	24.5	0.39
Back	6	8.7	13	3.8	19	4.7	
Chest	4	5.8	18	5.3	22	5.4	
Limbs	39	56.5	190	56.1	229	56.1	
Combination	7	10.1	31	9.1	38	9.3	
Types of treatment							
Monitoring and internal medical treatment	36	52.2	170	50.2	206	50.5	0.09
Maxillofacial surgery	1	1.5	4	1.2	5	1.2	0.80
Bone combination surgery	8	11.6	63	18.6	71	17.4	0.16

	Mean	SD	Mean	SD	Mean	SD	p-value
Length of hospital stay (days)	7.8	4.2	7.5	3.9	7.5	4.0	0.60
EQ-VAS	0.66	0.15	0.66	0.27	0.66	0.26	0.71
EQ—5D—5L index	0.35	0.38	0.37	0.37	0.37	0.37	0.80
Psychological distress	3.80	3.34	2.53	2.33	2.75	2.57	0.01

Table 3 shows the sleep quality of respondents experiencing traffic accidents. About 16.9% of respondents (n = 69) had self-rated sleep quality from fairly poor to poor. 50.7% of respondents (n = 207) slept lower than 5 h. Habitual sleep efficiency over 85% accounted for 81.3% (n = 318). The majority of participants did not use medicine for sleeping during the previous month. Approximately one-third of participants felt sleepy when doing daytime activities such as working, driving, or eating. Notably, 11.5% of respondents (n = 47) reported they felt tired and sleepy before driving on the day of suffering from accidents.

**Table 3:** Sleep quality of traffic accident patients.

Characteristics	n	%
Total	408	100
Self-rated sleep quality		
Very good	27	6.6
Fairly good	312	76.5
Fairly poor	64	15.7
Very poor	5	1.2
Sleep duration		
> 7 h	5	1.2
From 6 to ≤ 7 h	29	7.1
From 5 to ≤ 6 h	167	40.9
≤ 5 h	207	50.7
Habitual sleep efficiency (percentage)		
> 85%	318	81.3
75–84%	46	11.8
< 65%	27	6.9
Taking medicine for sleeping		
Not during the past month	393	96.3
Less than once a week	5	1.2
Once or twice a week	6	1.5
Three or more times week	4	1
Daytime dysfunction (score)		
0	272	66.7
1–2	115	28.2
3–4	19	4.7
5–6	2	0.5
Feeling tired, sleepy before driving on the day of the accident		
No	361	88.5
Yes	47	11.5

Table 4 reveals factors associated with sleeping problems among patients suffering from traffic injuries. Participants who were female were more likely to have poor sleep quality (OR = 0.51, 95% CI = 0.27; 0.96). Suffering from traumatic brain injury and was positively associated with an increasing score of daytime dysfunctions (Coef. = 0.58, 95% CI = 0.16; 1.00). Being comatose at hospitalization was related to poor sleep quality (Coef. = 4.49, 95% CI = 1.17; 17.22). In term of mental health, the increase of psychological distress score had a positive association with poor sleep quality (OR = 1.21, 95% CI = 1.08; 1.36), feeling tired or sleepy before joining traffic on the day of having accident (OR = 1.18, 95% CI = 1.05; 1.33) and higher score of daytime dysfunctions (Coef. = 0.24, 95% CI = 0.18; − 0.31).

**Table 4:** Factors associated with sleeping problems.

Characteristics	Sleep quality (sleep disturbance -1 vs normal sleep quality -0)		Feeling tired, sleepy before joining traffic on the day of the accident		Sleep duration		Habitual sleep efficiency (percentage)		Use of Sleeping medication		Daytime dysfunction (score)	
	OR	95% CI	OR	95% CI	Coef.	95% CI	Coef.	95% CI	Coef.	95% CI	Coef.	95% CI
Age group (< 30—ref)												
30 to < 45	1.29	0.42; 3.97	1.13	0.47; 2.75	0.09	− 0.06; 0.24	0.42**	0.10; 0.74				
45 to < 60	1.18	0.40; 3.48	0.28**	0.09; 0.84	0.09	− 0.06; 0.25	0.77***	0.42; 1.12				
≥ 60	2.48*	0.89; 6.91	0.74	0.25; 2.20	0.21***	0.05; 0.37	1.37***	0.96; 1.77				
Gender (Female—ref)												
Male	0.51**	0.27; 0.96			− 0.15***	− 0.26; − 0.05	− 0.23*	− 0.49; 0.03	− 0.97	− 2.35; 0.40		
Employment (Employed—ref)												
Others			0.41	0.13; 1.29								
Types of patients (In-patient—ref)												
Out-patient											0.42**	0.06; 0.79
Health status when hospitalized (Conscious—ref)												
Comatose	4.49**	1.17; 17.22										
Different injury categories (No-ref)												
Hand							− 0.43	− 0.98; 0.12	2.11	− 0.94; 5.17		
Traumatic brain injury			2.12*	0.99; 4.56							0.58***	0.16; 1.00
Oral and facial							− 0.37	− 0.88; 0.14			0.5	− 0.16; 1.17
Spinal cord injury							0.58	− 0.14; 1.30				
Chest			3.53	0.62; 20.13							0.66	− 0.24; 1.57
Fracture							0.26*	− 0.02; 0.54				
Type of treatment (No-ref)												
Bone combination surgery					− 0.10	− 0.24; − 0.04	0.57***	− 0.98; − 0.17	1.68*	− 0.28; − 3.64		
Psychological distress	1.21***	1.08; 1.36	1.18***	1.05; 1.33	0.04***	0.02; 0.06					0.24***	0.18; 0.31
EQ-5D-5L index (Unit: score)					0.1	− 0.04; 0.23	− 0.55***	− 0.92; − 0.18	2.74***	0.66; 4.81		

*** p < 0.01, ** p < 0.05, * p < 0.1. Adjusted for education, income quintile, living areas and EQ-VAS.

### Discussion

Our study highlighted critically low sleep duration and sleep quality among patients with road traffic accidents in Thai Binh, Vietnam. Being female, suffering from traumatic brain injury, being comatose at hospitalization and having higher psychological distress scores were associated with prevalence of sleep disorders. Empirical findings from our research suggest the importance of maintenance as well as improvement of sleep quality alongside clinical treatments and regular screening for sleeping disorders outside of the hospitals.

90% of patients with road traffic injuries sleep for less than 6 h a day, in which 50.6% sleep less than 5 h. Understandably, patients with medical conditions are more vulnerable to deterioration in sleep quality and quantity than their healthy peers, as indicated by various studies such as Orff et al.’s systematic review on traumatic brain injuries or Budh et al.’s cross-sectional study on patients with spinal cord injuries^42,43^. However, the average in the urban setting is critically lower than the average of the Vietnamese general population, which is 7 h and 14 min and is also among the least sleeping nations worldwide^44,45^. We attributed this gap to differences in access, delivery, plan of as well as budget for healthcare services between urban and central settings. As sleep quality falls under the mental healthcare category, a lack of mental health management will directly result in decay of sleep quality.

In small urban and less developed areas, mental healthcare is not a priority and rarely included in treatment plans^37,46^. A report of focus group discussions in Vietnamese health facilities highlighted several barriers to mental care access in urban areas of Vietnam^47^. On an administrative level, small urban facilities or district hospitals often lack mental medication supply, financial resources and inadequate workforce. Individually, people with mental disorders tend to not seek help due to lack of awareness on mental health issues or fear of causing burden to family. Such behavior could be attributed to lower education levels, lower budget for healthcare or dysfunctional family dynamics in urban areas. Above factors pose critical challenges for healthcare access even for physically healthy urban residents and are amplified among hospitalized patients.

Sleep quality was found to decrease with lower monthly household incomes, where the mean income of the good quality group was approximately 110 USD (2.5 million VND) higher than that of the poor-quality group. The average income of urban participants in this study was 388 USD (8.8 million VND). Meanwhile, the mean charge of hospitalization for moderate traumatic injury in Vietnam was 1400 USD (31.9 million VN. Although the national health insurance plan typically covers 80% of hospital costs, Vietnamese trauma patients will still suffer from a 277 USD (6.3 million VND) out-of-pocket cost, not to mention non-clinical expenditures such as accommodation, food and travelling^48,49^. The association between financial worries and insomnia has been confirmed in various studies, including Hall et al.’s on midlife women, Peltz et al.’s on American college students, or most recently in Saalwirth’s study among COVID-19 patients^50–52^. Moreover, unlike issues about healthcare service or treatment plan, the financial burden posed by traffic accidents is present long-term depending on the loss of productivity due to each injury. Therefore, longitudinal studies and follow-ups should be conducted to assess sleep quality in discharged patients and provide interventions accordingly.

Psychological distress was also associated with feeling tired and sleepy before joining traffic on the day of the accident. This finding is in line with the majority of studies on road traffic accident. In a report on 1828 traffic crashes in UK in the course of 6 years, 17% of them were sleep-related^53^. Although no systematic report has been conducted on Vietnamese population, drowsy driving has been the cause of numerous accidents, especially in urban areas^54,55^. Moreover, sleeping medication is often used widely and unprescribed among occupational drivers to meet strict work requirements, such as night driving or restless workdays^56^. Although, the short-term use of sleeping pills could improve sleep quality and extend sleep duration^57^. However, several side effects of sleeping pills are mentioned such as it can make people unsteady, dizzy, forgetful, and hard to concentrate^58^. These side effects may increase your risk of having accidents and falls^58^. In addiction, with long-term use, sleeping pills become less effective over time, especially for people who are irresponsible and abuse of sleeping pills^58^. In our study, extensive use of sleeping medication and lower habitual sleep efficiency were both linked to psychological distress, an underlying cause of sleep deterioration. Therefore, a preventative approach to road traffic injuries should be emphasized. Vietnam should consult strategies adopted by developed countries, where effective screening models and intensive public health measures have been found to significantly reduce road traffic accidents. For example, a driver fatigue campaign called “Stop, Sip, Sleep” since 2015 provided a guideline of 4 steps to relieve sleepiness among drivers in Ireland, or a Driver Fatigue Awareness Campaign by the International Association of Oil and Gas Producers has aimed at raising awareness through training workshops and as well as providing guidance for companies and safety managers worldwide^59,60^. Or European Union (eg. 2014/85/EU) directive to prevent patients with sleepiness untreated driving from continuing to drive until the disorder is effectively treated and physician certification is required to confirm suitability to continue driving^61,62^.

We propose following the implications from above results. First, maintaining and improving sleeping quality and psychological distress should be included in standard traumatic injury treatments, especially in urban health facilities. Globally and in more developed health facilities of Vietnam, sleep quality has been increasingly regarded as an integral indicator of treatment outcome and should therefore also be adopted by small urban facilities. Secondly, screening for sleeping disorders should be conducted regularly, not only in the treatment and follow-up periods after injury but also in the prevention process, such as daytime running lights, fluorescent jackets, and driver training to reduce road sleepiness^63–66^. Regulations on purchase of sleeping pills should be implemented to reduce unprescribed and irresponsible use of medication. Thirdly, as psychological distress due to financial issues contributes greatly to deterioration of sleep quality, healthcare managers should consider a systemic re-allocation of resources to optimize service and reduce unnecessary costs for patients. Ultimately, this study serves as exploratory research in a field with relatively little evidence in Vietnam, which is sleep quality among patients with road traffic injuries. Future studies under this umbrella topic can follow several pathways: assessing sleep quality in different types of road traffic accidents/vehicles, comparing sleep quality of patients with road traffic injuries to that of patients with the same injuries but not due to road traffic injuries, or investigating non-clinical determinants such as strong emotional circumstances like injury of a close person.

Certain limitations should be considered in this study. As we adopted the cross-sectional study model, causal relationships between variables were only hypothesized and could not be entirely proven. Second, under- or over-estimation of answers could occur due to recall bias and social desirability. Moreover, the convenience sampling technique limited the ability to generalize our findings to the whole Vietnamese population. Therefore, further studies should be conducted in different databases and a diverse range of patient populations, especially in-depth and longitudinal research directions to improve the capacity of generalizability the results.

## Conclusions

Our study revealed severe deterioration of sleep quality among patients with road traffic accidents in Thai Binh, Vietnam. Besides gender and specific injury characteristics, income level and psychological distress associated with use of sleeping pills were found to be determinants of poor sleep quality. Main implications regarded integration of sleep quality in treatment procedure and assessment, sleep disorders screening, management of sleep medication and redistribution of resources. In-depth and longitudinal research directions were proposed from this exploratory study.

## Data Availability

The datasets used and/or analysed during the current study are available from the corresponding author on reasonable request.

## Abbreviations

HRQOL: Health-related Quality of Life
EQ-5D-5L: EuroQol 5 Dimensions—5 levels scale
PSQI: The Pittsburgh Sleep Quality Index
